# Optimization and Microstructural Studies on the Machining of Inconel 600 in WEDM Using Untreated and Cryogenically Treated Zinc Electrodes

**DOI:** 10.3390/ma16083181

**Published:** 2023-04-18

**Authors:** Satyanarayana Kosaraju, Phaneendra Babu Bobba, Surender Reddy Salkuti

**Affiliations:** 1 Department of Mechanical Engineering, Gokaraju Rangaraju Institute of Engineering and Technology, Hyderabad 500090, India; 2 Department of Electrical and Electronics Engineering, Gokaraju Rangaraju Institute of Engineering and Technology, Hyderabad 500090, India; 3 Department of Railroad and Electrical Engineering, Woosong University, Daejeon 34606, Republic of Korea

**Keywords:** WEDM, Inconel, microstructural study, optimization, SEM, regression model

## Abstract

Any industry that manufactures dies, punches, molds, and machine components from difficult-to-cut materials, such as Inconel, titanium, and other super alloys, largely relies on wire electrical discharge machining (WEDM). In the current study, the effect of the WEDM process parameters on Inconel 600 alloy with untreated zinc and cryogenically treated zinc electrodes was investigated. The controllable parameters included the current (IP), pulse-on time (Ton), and pulse-off time (Toff), whereas the wire diameter, workpiece diameter, dielectric fluid flow rate, wire feed rate, and cable tension were held constant throughout the experiments. The significance of these parameters on the material removal rate (MRR) and surface roughness (Ra) was established using the analysis of the variance. The experimental data acquired using the Taguchi analysis were used to analyze the level of influence of each process parameter on a particular performance characteristic. Their interactions with the pulse-off time were identified as the most influential process parameter on the MRR and Ra in both cases. Furthermore, a microstructural analysis was also performed via scanning electron microscopy (SEM) to examine the recast layer thickness, micropores, cracks, depth of metal, pitching of metal, and electrode droplets over the workpiece surface. In addition, energy-dispersive X-ray spectroscopy (EDS) was also carried out for the quantitative and semi-quantitative analyses of the work surface and electrodes after machining.

## 1. Introduction

Advanced engineering materials and super alloys are integrated into regular applications due to the fact of their excellent mechanical properties. They possess a high strength-to-weight ratio, high hardness with excellent corrosive resistance, and nonreactive metal [1]. This superior quality leads to limitations in machining in traditional machining operations. In fact, because of their difficult-to-cut characteristics, they are difficult to machine in a conventional manner. Nontraditional machining (NTM) operations are being employed for the machining of these hard-to-cut metals, alloys, and superalloys. Laser cutting (LC), electrical discharge machining (EDM), wire cut electric discharge machining (WEDM), and chemical etching (CE) have been employed in the practice of machining of these hard-to-cut metals and super alloys [2,3]. Thus, they have been used in the machining of advanced equipment and even biomedical devices [4]. WEDM is one such process that works on the principal of the phenomena of the electrical conductivity of metal and the polarization of electrodes and cathodes. It uses the special technique of cutting the metal via the sparks created between the workpiece and electrode (i.e., wire form). These sparks erode the material, and the di-electric fluid acts as an eroded metal carrier [5]. The IP, voltage (v), Toff, Ton, and flow of dielectric fluid are the parameters that play major roles in the WEDM process. This process is proven to be one of the most cost effective, shortening the machining duration and resulting in an appreciable surface finish. WEDM has been found to be a precision machining tool while working on NIMONICS C263 super alloys [6]. Strake et al. [7] empirically studied and indicated through their findings that by using WEDM, it is possible to obtain the accuracy and dimensions within the tolerance limit. Under extreme machining circumstances, an investigation of an EN X30WCrV9-3 tool steel using a CuZn37 wire electrode revealed a variance of 2 µm to 25 µm. Regardless of the geometry or geometries of the finished product, WEDM may be simply used to machine any required shape. One such example is its effectiveness in producing items such as noncircular gears [8] of complex shapes and metals, which are electrically conductive in nature. Rakesh et al. [9] reported that electrode traces were found over the NITINOL super alloy when machining with molybdenum wire. Upon SEM and EDS studies, they determined that there were impurities over the base metal. The rate of electrode wear and MRR are dependent variables, which depend on the rate of electrical conductivity; that is, metals with a higher rate of electrical conductivity show a higher rate of material removal rate. Sachin and kulkarni [10] observed that the surface roughness, material removal rate, and overcut increase with an increase in the peak current and pulse-on time, and it decreases with a decrease in the voltage, pulse-off time, and wire feed rate during the WEDM of NIMOMIC-75. In the WEDM process, the rapid electrode wear rate and low material removal rate are challenging tasks to overcome. These challenges can be overcome by coating and cryogenically treating the electrode before machining. In the past, copper wire was used as the electrode [11], which has been replaced with zinc and molybdenum due to the fact of advancements in the engineering of wire electrodes, as they are processed with composites coated over the base metal. It was reported that composite coatings [12] improve the conductivity and are flushable and accurately cut; in addition, they have a low tool wear rate and less weldability over other metals. Advancements in these wires not only improve the machinability but no longer restrict them to single use, thereby increasing the rate of production and also decreasing the machining cost. A few studies have shown that WEDM is also used in the machining or cutting of CFRP-laminated composites [13]. Experimental studies were carried out on Inconel 718 using a zinc-diffused electrode wire. Luca et al. [14] performed WEDM to determine the surface integrity of Inconel 718. The researchers performed the machining using uncoated brass wire and zinc-coated copper wire. They concluded that there was a 35% increase in the wire feed rate along with a 40% reduction in wire consumption using coated wire compared to uncoated wire. Zei Chen [15] carried out an investigation on improving the characteristics of WEDM, and a comparison was made between copper wire and zinc-coated WEDM, and a 21.18% reduction in the wire wear rate along with a 16.67% improvement in the surface roughness were reported. Ruma et al. [16] discussed the effects of three wire electrodes composed of plain brass wire, silver-coated brass wire, and zinc-coated wire on the machining of maraging 300 steel. They concluded that the zinc-coated wire proved to be optimum in terms of microcracks and surface integrity, and the silver-coated wire showed better quality surface with high-speed machinability.

Cryogenic treatment is a branch of engineering that focuses on the treatment of metal or material at subzero temperatures in order to change its mechanical properties. It ranges from 100 k to almost an absolute zero temperature of −273 °C. Cryogenic treatment involves three principal stages: cooling, soaking, and warming. The cooling period is the duration of time where the material is cooled from room temperature, or finial temperature, to the subzero temperature. The faster the cooling period, the more defects are observed. Cryogenic treatment will reduce the adhering properties and the micropores in the metals due to the elements’ dislocation and shrinkage, which reduces the internal spaces. This causes a high and efficient flow of electrons through them, with high bonding between the shrunken elements, leading to a reduction in the rate of the metal element’s erosion. Generally, most composite metals or alloys experience a change in their mechanical properties, such as Youngs’s modulus, hardness, stiffness, shear modulus, rigidity, and bulk modulus, from cryogenic treatment [17]. When looking at the impact of deep cryogenic treatment on the corrosion properties of different high-speed steels, Jure et al. [18] found that the corrosion properties and hardness both improved after deep cryogenic treatment. Engin et al. [19], in their research on a shallow and deep cryogenically treated WEDM process, stated that cryogenic treatment not only increased the hardness but also improved the machinability. They concluded that a high electric conductivity was observed in deep cryogenic treatment compared to shallow cryogenic treatment. Furthermore, the MRR and surface roughness were improved after the cryogenic treatment. Kogbara et al. [20] reported that a faster cooling rate of metal leads to defects, such as thermal cracks, irregular grain size, and micropores in the material bonds. They suggested that the slower the cooling rate, the better the cryogenic effect. Dhande et al. [21] reported, in their study on tungsten carbide, that there is a greater influence of the soaking time on metals. Tahir et al. [22] discussed that the use of the WEDM process on HSLA proved that cryogenic treatment improves the electrical conductivity, thereby increasing the material removal rate and decreasing the surface roughness.

The soaking period is the duration of time when the actual cryogenic process takes place, for example, metals set to be liquid-cooled or air-cooled and maintained in subzero temperatures for a duration of time as per standards. During this stage, there is a change in bonding, or the moment when atoms move to another location. Liquid dip cooling may also cause surface hardness [23]. For example, liquid cooling with the aid of liquid carbon dioxide or liquid nitrogen causes surface hardening by the carbonization or nitration effect on the surface. In order to minimize this, metals are usually coated or sealed, or sometimes exposed to cool-air-type cryogenic treatment. Gill et al. [24] discussed the soaking period and its effects on metals. The warming period refers to returning the cooled metal to normal room temperature. The limitations are the same as for the cooling period. The rate of change in the temperature is equal to the finer-grained size of the metal. Corruccini [25] studied the properties and their effects of cooling on metals. Studies have also been carried out on Inconel 718 dielectric fluid mixed with ceramic powder using a copper wire that underwent cryogenic treatment as an electrode. Kapoor et al. [26] examined the impact of deep-cryogenic-treated brass wire electrodes. The experimental findings demonstrate that deep cryogenic treatment refines the structure more than noncryogenic therapy. The performance of wire electrical discharge machining was also investigated, as well as the impact of deep cryogenic treatment on brass wire electrodes. The most effective parameters for the maximum material removal rate were investigated using a Taguchi experimental design [27]. Asarudheen et al. [28] investigated how di-electric fluid also plays a role in machinability. They concluded that in an EDM process that mixes different powders into dielectric fluids, there is an increase in the efficiency in terms of an increase in the metal removal rate, a reduction in the tool wear, and an improved surface quality. Jay et al. [29] performed a near-dry and low-dielectric-fluid WEDM process over nitinol SMA (nickel titanium alloy) using molybdenum wire as an electrode.

Stefan et al. [30] discussed the working conditions and performance of Inconel 617 during the WEDM process using brass wire as an electrode. Usman et al. [31] investigated the production of recast layers during Ti-6Al-4V WEDM machining with varied diameters of brass wire. Furthermore, it was estimated that a 0.15 mm wire diameter improves the surface roughness by 25%. Asgar et al. [32] discussed the multiresponse optimization using an RSM-GRA analysis performed on pure titanium using WEDM with a molybdenum wire with a diameter of 0.18 mm, and they found that a Ton of 6 µs, Toff of 4 µs, and current of 6A were found to be the optimum conditions for the maximum MRR and minimum SR. Jin [33] used a reliable multiobjective optimization process based on a Gaussian regression process and concluded that the optimum set of process parameters to maximize the material removal rate and minimize the surface roughness were a Ton of 6 µs, Toff of 4 µs, and discharge current of 6 A during the machining of chromium alloy with molybdenum wire with a diameter of 0.17 mm in the WEDM process. Bandar et al. [34] discussed the microstructural characterization and micromechanical properties of the recast layer that took place during the WEDM of Inconel 718 alloy. They reported that the machined surface showed the presence of depressions, shallow cracks, and resolidified materials. Furthermore, the thickness of the recast layer was approximately 6.2 µm. Pawan Kumar et al. [35] conducted a microstructural analysis and the multiresponse optimization of the WEDM of Inconel 825 using RSM. They concluded that 110 machine units of pulse-on time, 35 machine units of pulse-off time, a 46-volt gap voltage, a 120 ampere peak current, 11 machine units of wire tension, and a 5 m/min wire feed are recommended to obtain the maximum material removal rate and minimum surface roughness and wire wear ratio. In addition, they also concluded that under these conditions, relatively fewer craters, pockmarks, cracks, and pulled-out material were found on the wire electrodes and work specimen surfaces. Benes et al. [36] experimentally showed that a surface machined using WEDM experiences surface hardness, a change in the elemental characteristics of the subsurface of the recast layer, and a tendency to become a heat-affected zone. Because of the surface’s impurities and fractures, surfaces that undergo WEDM are likely to have a high level of corrosive adoptability. It has been demonstrated by research on AISI 316Ti and AISI 303 in pyrolysis experiments that both metals display significant corrosion and oxidation after 28 days. Sharma et al. [37] proposed that a smaller diameter wire enhances productivity, as well as the surface quality, of machined components compared to larger diameter wires. Furthermore, smaller diameter wires have demonstrated a lower recast layer thickness, less hardness alteration, and a quicker manufacturing time when compared to larger wire diameters. Katerna et al. [38] concluded that there was a change in the residual stress of the metal’s subsurface caused by spark erosion and electrode welding over the machined surface. The authors also observed a change in the concentration of Al, Fe, and O on the subsurface of the machined surface of Ti-6Al-4V, as well as cracks on the subsurface caused by spark erosions. Sergey et al. [39] used graphical and pictorial representations to show the extent of the heat-affected zone at the recast depth in their study on hard alloy. While using WEDM, the paper also illustrated the erosion products and vibration spectrum.

In the present research work, the optimization of the process parameters in WEDM with cryogenically treated zinc electrodes and untreated electrodes in the machining of Inconel 600 was carried out, and the results were compared. Furthermore, microstructural studies were performed to study the recast layer thickness, micropores, cracks, pitching of metal, and electrode droplets over the workpiece surface. In addition, energy-dispersive X-ray spectroscopy (EDS) was performed to evaluate the work surface and electrodes after machining, both quantitatively and semi-quantitatively.

## 2. Materials and Methods

In the present work, investigations were carried out on Inconel 600 alloy that was 40 mm in diameter with an electrode that was 0.2 mm in diameter, and cryogenically treated and untreated conditions were employed in the machining. Heat treatment was carried out on the work material at a temperature of 500 °C for 3.5 h, and the furnace was cooled to remove the internal stresses that are induced while forming, rolling, or tuning during the preparation of the material. Zinc-coated wire electrodes were cryogenically treated with liquid nitrogen at a temperature of −100 °C for a soaking period of 5 h. After the treatment, the electrical conductivity of the electrode was calculated in accordance with the International Annealed Copper Standard (IACS), a 33% increase in the electrical conductivity was found with the treated electrode and a 27% increase with the untreated electrode.

**Figure 1:**
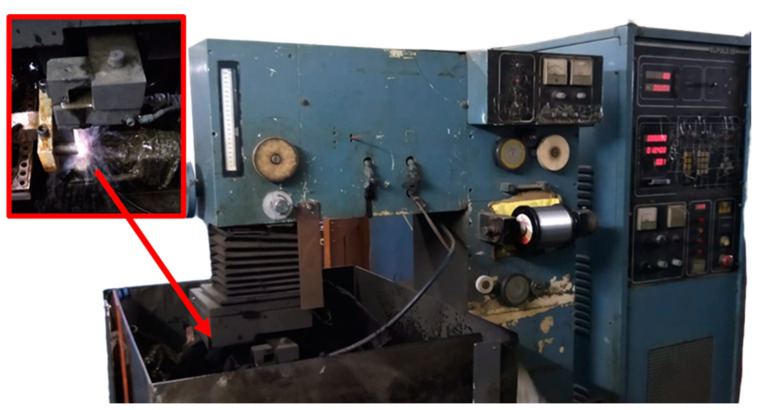
Experimental setup.

Orthogonal arrays based on the design of experiments (DoE) were used to carry out the experiments, because when using a full factorial design, the number of experimental runs grows exponentially as the number of components and their values increase. These findings necessitate extensive testing that costs a lot in terms of expenditure and time. With a small number of experimental runs, it is thought to be possible to balance these two negative aspects while also looking for the ideal process condition. Therefore, a total of 9 experiments based on Taguchi’s L9 orthogonal array, each having a combination of different levels of the factors, as shown in Table 1, were carried out. A high-precision WEDM ELPULS-20 maxi-cut of an Electra make was used for the experimentation (Figure 1). The responses measured were the material removal rate and surface roughness.

The material removed during the WEDM (i.e., in terms of mass) was measured by the difference in weight before machining and after the machining-to-machining time. Thereafter, the experimental MRR was calculated for all experiments with the cryogenically treated electrodes and untreated electrodes. Moreover, a surf-test SJ320 roughness measurement device was used to measure the surface roughness, with a cut-off length of 0.8 mm. Table 2 shows the input parameters and the results of the experiments machining the Inconel 600 with the untreated and cryogenically treated electrodes.

## 3. Results

### 3.1. S/N Ratio Analysis

In the Taguchi approach, the terms “signal” and “noise” stand for the desired value (mean) and the unwanted value, respectively, for the performance characteristic. Taguchi measures the quality attribute deviating from the desired value using the S/N ratio. Many S/N ratios, such as higher-the-better, lower-the-better, and nominal-the-better, are available depending on the type of characteristics. The S/N ratio, *η*, is defined as,

*η* = −10 log (mean-square deviation) (1)

For any machining process, smaller Ra values and higher MRR values are often preferred. The current work, therefore, chose smaller as the better Ra criterion and higher as the better MRR criterion. The mean square deviations (MSDs) for the smaller-the-better quality (2) and the higher-the-better quality (3) feature are written as,

(2) $$MSD=\frac{1}{x}\overset{n}{\underset{i=0}{\sum }}y_{i}^{2}$$

(3) $$MSD=\frac{1}{x}\overset{n}{\underset{i=0}{\sum }}\frac{1}{y_{i}^{2}}$$

where *x* is the number of tests, and *y_i_* is the value of the MRR and Ra of the *i*th test.

Using Equations (1)–(3), Table 2 displays the experimental data for Ra, MRR, and the appropriate S/N ratios (2) and (3). The influence of each cutting parameter may thus be separated at various levels because the experimental design is orthogonal. By averaging the S/N ratios for experiments 1–3, 4–6, and 7–9, respectively, it is possible to determine the mean S/N ratio for the current at levels 1, 2, and 3. Table 3 and Table 4 show the results of a comparable calculation for the mean S/N ratio for all trials, which implicates cutting Inconel 600 with both the untreated and cryogenically treated electrodes. The higher the S/N ratio, regardless of whether the quality characteristic is lower or higher, the more closely the output characteristics follow the desired value. It can be found from Table 3 and Table 4 that the optimum combination to maximize the MRR with the treated and cryogenically treated electrodes is IP at level 2 (i.e., 5 amps), Ton at level 1 (i.e., 2 micro sec), and Toff at level 3 (i.e., 9 micro sec) and in order to obtain a good surface finish is IP at level 3 (i.e., 7 amp), Ton at level 1 (i.e., 2 micro sec), and Toff at level 1 (i.e., 5 micro sec).

### 3.2. Analysis of Variance

Sir Ronald Fisher introduced the analysis of variance (ANOVA) [40]. The degree of confidence for this analysis was 95%. ANOVA was used to determine which process parameter had a noticeable impact on the performance parameters. Table 5 and Table 6 show the results of ANOVA for the untreated electrode (MRR and Ra) and cryogenically treated electrode (MRR and Ra). It was found that Toff and the interaction of Toff parameters affected the MRR and Ra in both the cases of the untreated and cryogenically treated electrodes. It also confirms from Table 3 and Table 4 that Toff ranked first in all cases.

### 3.3. Microstructural Analysis

The microstructure under the optimum condition was evaluated, and the WEDM samples of the Inconel 600 machined with an untreated and treated electrode fractured surfaces were studied. The surface features of the work material and electrode were analyzed using a scanning electron microscope (SEM) (EVO 18 Zeiss) and the EDS (EDAX EDS system element fixed model) test for the qualitative and semi-quantitative study of the chemical composition.

#### 3.3.1. SEM/EDS Examination of the Work Surfaces

**Figure 2:**
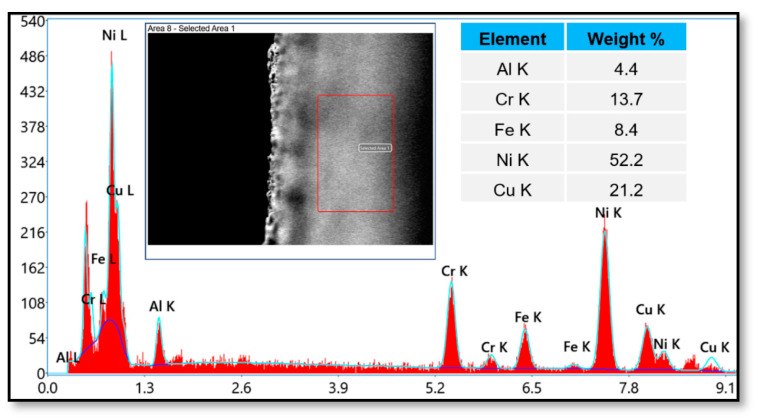
SEM and EDS of an Inconel 600 specimen before machining.

Specimens of approximately 10 mm × 5 mm were prepared using WEDM. The specimens were then cleaned in an acetone-infused ultrasonic bath. The specimens were then dried using an air-blaster in preparation for further SEM/EDS examinations. Figure 2 shows the SEM micrographs and EDS analysis of the sample where the machining was not performed, in other words the analysis was performed on the base metal of Inconel 600, which showed no traces of zinc in the analysis. This demonstrates that the metal chosen was free of zinc elements and confirmed that the base metal was a nickel (Ni) super alloy.

**Figure 3:**
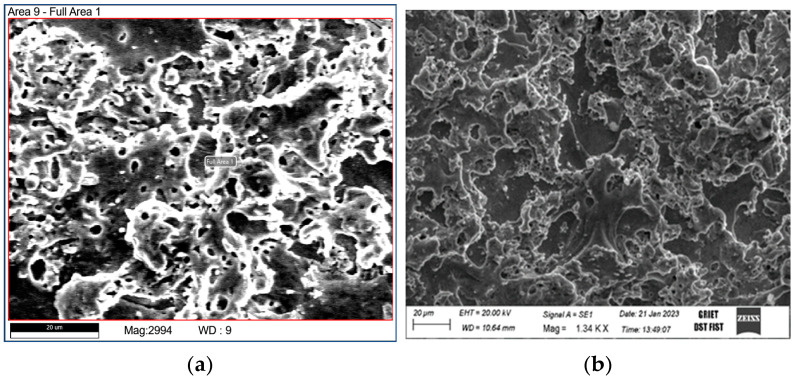
Micrographs of the machined surfaces of the (**a**) untreated and (**b**) cryogenically treated electrodes.

Figure 3a shows SEM micrographs of a machined surface with an untreated and treated zinc wire electrode. The deep craters and many gas holes on the surface were found to be typical topography produced by the melting and recasting process. The micrograph of the specimen after the electrode had been cryogenically treated, however, was very different from the aforementioned findings. Figure 3b shows a matte surface with small nodules; this may be related to the treated electrode’s fine-grained structure. Materials of the electrode (zinc) were detected with EDS analysis, as shown in Table 7, for both the treated and untreated electrodes. Furthermore, it can be observed from the table that the decrease in the presence of zinc decreased from 8.3% (untreated) to 4.8% (treated) may be due to the increase in the electrical conductivity of the material.

**Figure 4:**
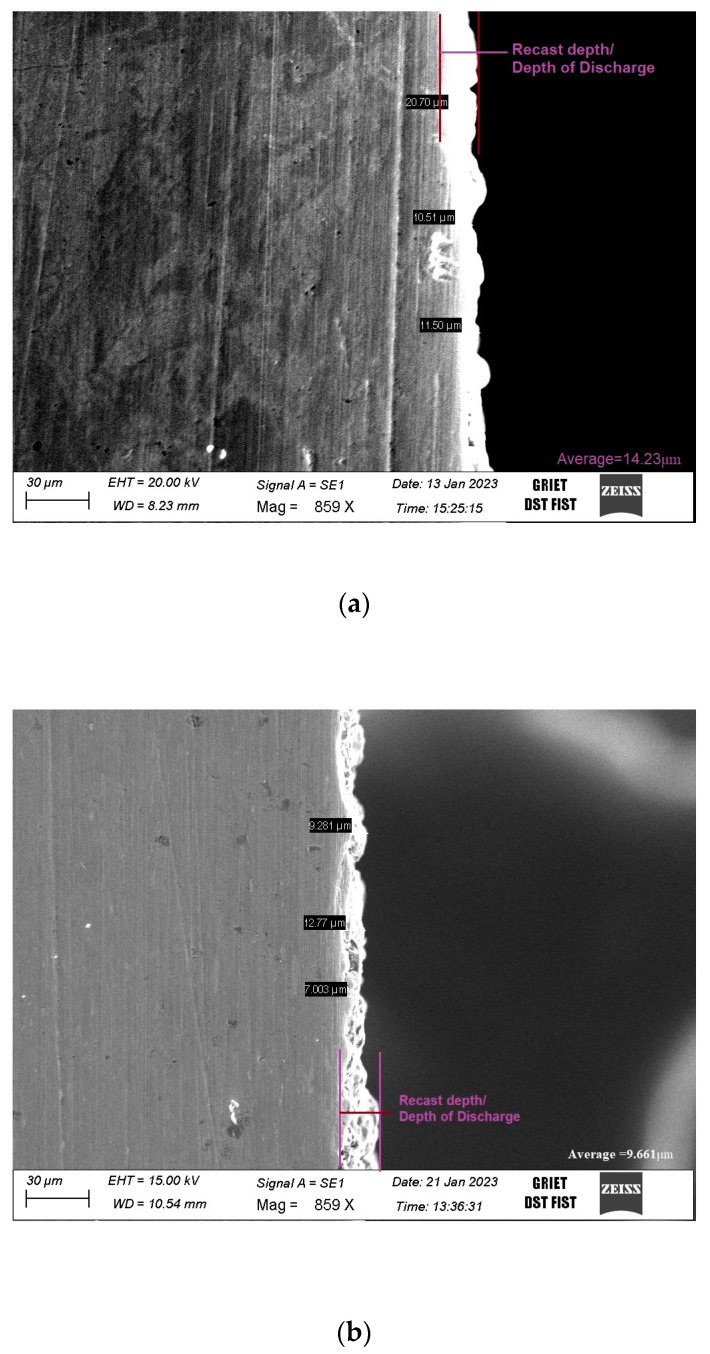
Recast layer on work surface with various electrodes. (**a**) Untreated electrode. (**b**) Treated electrode.

With further investigation of the zinc content on the machined Inconel surface, a microstructural analysis was carried out for the recast depth. The recast depth is the amount of depth to which the etching or erosion takes place, and it is viewed at the cross-sectional area of the machined surface [41]. The recast depth for the untreated electrode was observed to be on average 14.23 μm, and with the treated electrode it was 9.661 μm, as shown in Figure 4. This may be due to the low zinc content, leading to a lower depth of the metal weld. From the micrographs, it can also be observed that the irregular depth profile and weld up burrs on the surface were greater in the case of the untreated sample when compared with the treated sample.

#### 3.3.2. Examination of the Electrode with SEM

**Figure 5:**
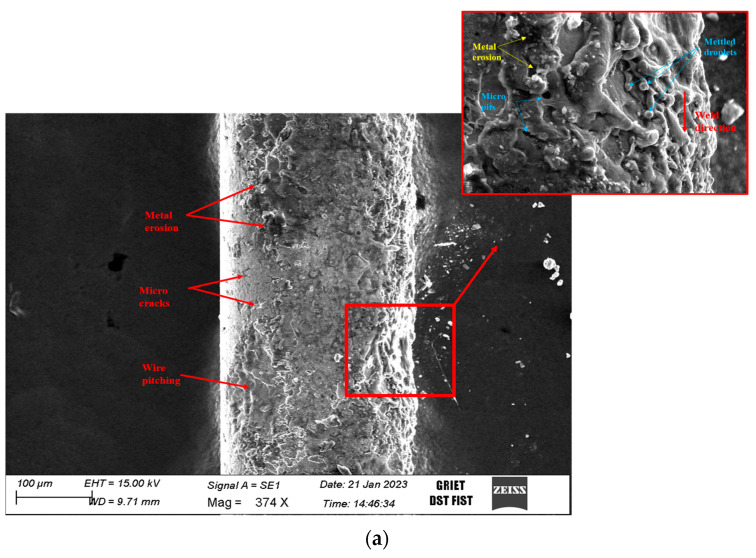

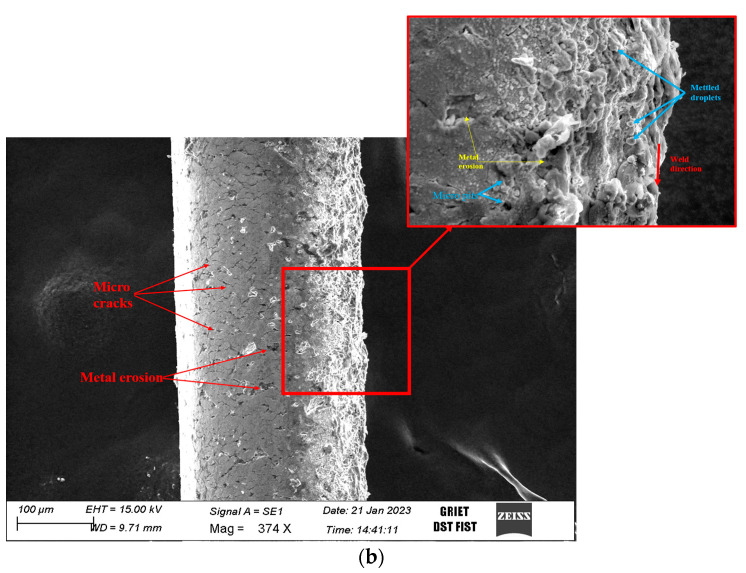
Work surface machining with various electrodes. (**a**) Untreated electrode. (**b**) Treated electrode.

The recast depth, MRR, and Ra are dependent on the electrode pitching and wear rate. The depth of the recast and amount of zinc welded over the surface were directly proportional to the electrode wear rate, as shown in Figure 5. The rate of tool wear also depends on the rate of electrical conductivity and electric polarity. In view of this, a detailed study was performed on the untreated and treated electrodes using SEM. The metal erosion, microcracks, electrode pitching, and molten metal droplets were microstructurally examined on the electrodes after the machining in both cases. Cryogenic treatment and quenching of the electrodes resulted in grains fine in size and with a uniform orientation. Due to the fact of this fine structural arrangement and bonding, the element’s erosion was low in the case of the treated electrode. From the micrographical images, with the pitching of the electrode, minor cracks were observed. Figure 5a represents the microcracks and pitching of the untreated electrode in which microcracks were observed to be more numerous and the pitching of the material higher compared to Figure 5b, i.e., the cryogenically treated electrode. The quenching of the electrode resulted in grains that were fine in size and with a uniform orientation. Due to the fact of this fine structural arrangement and bonding, the element’s erosion was low in the case of the treated electrode.

## 4. Conclusions

In the current work, the optimization of WEDM process parameters on Inconel 600 alloy with untreated zinc and cryogenically treated zinc electrodes were analyzed experimentally, and microstructural characterization was performed. Some of the key findings are stated below.

- In WEDM, the use of IP at 5 amps, Ton at 2 micro sec, and Toff at 9 micro sec is recommended to obtain a higher MRR in untreated and treated conditions;
- In WEDM, the use of IP at 7 amp, Ton at 2 micro sec, and Toff at 5 micro sec is recommended to obtain the minimum Ra in untreated and treated conditions;
- Through ANOVA, the Toff and the interaction of Toff parameters are the parameters that most affect the MRR and Ra in untreated and treated conditions;
- The recast depth for the untreated electrode was observed to be on average 14.23 μm, and with treated electrode it was 9.661 μm;
- The bonding of the elements in the case of the treated electrode improved due to the fact that the cryogenic treatment leads to a reduction in the number of pores and microcracks compared to the untreated electrode;
- The untreated electrode had high electrode erosion and pitching on the surface compared to the treated electrode.

The results are confined to the range of parameters; however, the study can be expanded by considering other characteristics, such as wire feed, wire diameter variation, wire tension, and application-specific parameters.

## Figures and Tables

**Table 1:** Process parameters and their levels.

S. No	Parameters	Symbol	Units	Level 1	Level 2	Level 3
1	Current	I	A	3	5	7
2	Pulse-on time	Ton	micro sec	2	4	6
3	Pulse-off time	Toff	micro sec	5	7	9

**Table 2:** Experimental results for the untreated and cryogenically treated electrodes and the corresponding S/N ratio.

S. No	IP	Ton	Toff	Untreated							Cryogenically Treated						
				Weight (g)		Time (min)	MRR (g/min)	S/N Ratio MRR	Ra (µm)	S/N Ratio Ra	Weight (g)		Time (min)	MRR (g/min)	S/N Ratio MRR	Ra (µm)	S/N Ratio Ra
				Before	After						Before	After					
1	3	2	5	177.25	158.24	43	0.442	−7.09	1.31	−2.37	240.12	222.00	41	0.442	−7.09	1.29	−2.23
2	3	4	7	158.24	140.98	48	0.360	−8.87	1.92	−5.69	222.00	205.42	45	0.368	−8.68	1.75	−4.84
3	3	6	9	140.98	121.20	43	0.460	−6.74	3.03	−9.61	205.42	186.30	42	0.455	−6.84	2.91	−9.27
4	5	2	7	121.20	103.45	41	0.433	−7.27	2.72	−8.70	186.30	167.80	40	0.463	−6.69	2.11	−6.47
5	5	4	9	103.45	82.95	38	0.539	−5.36	3.46	−10.78	167.80	148.22	36	0.544	−5.29	3.37	−10.54
6	5	6	5	82.95	64.80	37	0.491	−6.18	1.45	−3.25	148.22	130.12	38	0.476	−6.45	1.31	−2.33
7	7	2	9	64.80	46.78	36	0.501	−6.00	1.49	−3.46	130.12	112.54	33	0.533	−5.47	1.34	−2.53
8	7	4	5	46.78	26.58	45	0.449	−6.95	1.32	−2.41	112.54	94.99	38	0.462	−6.71	1.27	−2.08
9	7	6	7	26.58	17.01	35	0.274	−11.24	1.99	−5.99	94.99	86.75	32	0.258	−11.77	1.78	−4.98

**Table 3:** S/N ratio response for the untreated electrode.

Level	MRR			Ra		
	IP	Ton	Toff	IP	Ton	Toff
1	−7.57	−6.79	−6.75	−5.89	−4.84	−2.68
2	−6.27	−7.07	−9.14	−7.58	−6.29	−6.79
3	−8.07	−8.06	−6.04	−3.95	−6.28	−7.95
Delta	1.80	1.27	3.10	3.62	1.45	5.27
Rank	2	3	1	2	3	1

**Table 4:** S/N ratio response for the cryogenically treated electrode.

Level	MRR			Ra		
	IP	Ton	Toff	IP	Ton	Toff
1	−7.53	−6.42	−6.75	−5.44	−3.74	−2.21
2	−6.14	−6.89	−9.05	−6.45	−5.82	−5.43
3	−7.99	−8.35	−5.87	−3.20	−5.53	−7.45
Delta	1.85	1.93	3.19	3.25	2.08	5.28
Rank	3	2	1	2	3	1

**Table 5:** Analysis of variance for the untreated electrode.

MRR							Ra						
Source	DF	Adj. SS	Adj. MS	F-Value	*p*-Value	% Con.	Source	DF	Adj. SS	Adj. MS	F-Value	*p*-Value	% Con.
Regression	6	0.049	0.008	8.56	0.108		Regression	6	4.388	0.731	1.88	0.387	
IP	1	0.010	0.010	10.8	0.081	12.32	IP	1	0.246	0.246	0.63	0.51	6.11
Ton	1	0.000	0.000	0.19	0.707	0.21	Ton	1	0.131	0.131	0.34	0.62	3.25
Toff	1	0.029	0.029	31.09	0.031	35.45	Toff	1	1.100	1.100	2.83	0.235	27.28
IP * IP	1	0.011	0.011	11.34	0.078	12.93	IP * Ton	1	0.857	0.857	2.2	0.276	21.25
Ton * Ton	1	0.001	0.001	0.53	0.543	0.60	IP * Toff	1	1.066	1.066	2.74	0.24	26.42
Toff * Toff	1	0.031	0.031	32.76	0.029	37.35	Ton * Toff	1	0.244	0.244	0.63	0.512	6.04
Error	2	0.002	0.001			1.14	Error	2	0.778	0.389			9.64
Total	8	0.051	0.083			100.00	Total	8	5.166	4.033			100.00

**Table 6:** Analysis of variance for the cryogenically treated electrode.

MRR							Ra						
Source	DF	Adj. SS	Adj. MS	F-Value	*p*-Value	% Con.	Source	DF	Adj. SS	Adj. MS	F-Value	*p*-Value	% Con.
Regression	6	0.056	0.009	4.65	0.188		Regression	6	3.971	0.662	1.83	0.394	
IP	1	0.011	0.011	5.41	0.145	13.12	IP	1	0.134	0.134	0.37	0.604	5.23
Ton	1	0.000	0.000	0.09	0.789	0.23	Ton	1	0.158	0.158	0.44	0.576	6.15
Toff	1	0.028	0.028	13.92	0.065	33.74	Toff	1	0.645	0.645	1.79	0.313	25.11
IP * IP	1	0.011	0.011	5.55	0.143	13.44	IP * Ton	1	0.542	0.542	1.5	0.345	21.08
Ton * Ton	1	0.001	0.001	0.4	0.59	0.98	IP * Toff	1	0.649	0.649	1.8	0.312	25.25
Toff * Toff	1	0.030	0.030	14.89	0.061	36.07	Ton * Toff	1	0.081	0.081	0.22	0.683	3.14
Error	2	0.004	0.002			2.42	Error	2	0.721	0.361			14.04
Total	8	0.060	0.083			100.00	Total	8	4.693	2.569			100.00

**Table 7:** EDS results for the untreated and treated electrodes.

Electrode	Element	Cr K	Fe K	Ni K	Cu K	Zn K
Untreated	Weight%	11.1	5.8	41.7	22.8	8.3
Treated	Weight%	14.3	8.5	54.2	17.7	4.8

## Data Availability

Not applicable.
